# Acetylation-Mediated Post-Translational Modification of Pyruvate Dehydrogenase Plays a Critical Role in the Regulation of the Cellular Acetylome During Metabolic Stress

**DOI:** 10.3390/metabo14120701

**Published:** 2024-12-12

**Authors:** Aishwarya Rajakumar, Sarah Nguyen, Nicole Ford, Gbenga Ogundipe, Ethan Lopez-Nowak, Olena Kondrachuk, Manish K Gupta

**Affiliations:** Division of Metabolic and Cardiovascular Sciences, Burnett School of Biomedical Sciences, College of Medicine, University of Central Florida, Orlando, FL 32827, USA

**Keywords:** acetylation, ischemia, pyruvate dehydrogenase complex, cardiomyocytes, post-translational modification, metabolism, ryanodine receptor 3, mitochondria

## Abstract

**Background:** Cardiac diseases remain one of the leading causes of death globally, often linked to ischemic conditions that can affect cellular homeostasis and metabolism, which can lead to the development of cardiovascular dysfunction. Considering the effect of ischemic cardiomyopathy on the global population, it is vital to understand the impact of ischemia on cardiac cells and how ischemic conditions change different cellular functions through post-translational modification of cellular proteins. **Methods**: To understand the cellular function and fine-tuning during stress, we established an ischemia model using neonatal rat ventricular cardiomyocytes. Further, the level of cellular acetylation was determined by Western blotting and affinity chromatography coupled with liquid chromatography–mass spectroscopy. **Results:** Our study found that the level of cellular acetylation significantly reduced during ischemic conditions compared to normoxic conditions. Further, in mass spectroscopy data, 179 acetylation sites were identified in the proteins in ischemic cardiomyocytes. Among them, acetylation at 121 proteins was downregulated, and 26 proteins were upregulated compared to the control groups. Differentially, acetylated proteins are mainly involved in cellular metabolism, sarcomere structure, and motor activity. Additionally, a protein enrichment study identified that the ischemic condition impacted two major biological pathways: the acetyl-CoA biosynthesis process from pyruvate and the tricarboxylic acid cycle by deacetylation of the associated proteins. Moreover, most differential acetylation was found in the protein pyruvate dehydrogenase complex. **Conclusions:** Understanding the differential acetylation of cellular protein during ischemia may help to protect against the harmful effect of ischemia on cellular metabolism and cytoskeleton organization. Additionally, our study can help to understand the fine-tuning of proteins at different sites during ischemia.

## 1. Introduction

Cardiovascular disease is one of the global leading causes of death. The most common behavioral risk factors for cardiovascular diseases include unhealthy lifestyles, lack of physical activity, smoking, and uncontrolled alcohol consumption [1]. Ischemic/coronary heart disease (IHD/CHD) can present as sudden cardiac death or acute myocardial infarction and is the most predominant of the cardiovascular diseases [2]. Ischemic heart disease occurs when blood flow [3] in the heart is restricted, often by the blockage of coronary arteries [4,5]. This can present clinically as a heart attack, arrhythmia, or heart failure [6]. Myocardial ischemia is primarily caused by atherosclerosis, which is plaque buildup on arterial walls that inhibits blood flow [5,6]. Blood flow carries oxygen to myocardial tissue, which is essential for the function of cardiac muscle fibers because they consume significant ATP [7]. Cardiomyocytes are rich in mitochondria that rely on aerobic metabolism for energy production and are therefore less efficient in utilizing anaerobic metabolic pathways during anoxic conditions, leaving them highly susceptible to ischemic conditions [8,9,10]. The search for therapeutic agents to act against ischemic heart disease can begin with the study of epigenomics [11]. Some CVD risk factors known to modify epigenetic markers are nutrition, smoking, pollution, stress, and the circadian rhythm [12,13]. Common epigenetic mechanisms include DNA methylation, histone modifications, and microRNA alterations, all of which enable the cell to respond to environmental changes [12]. They cause rearrangement of chromatin structure and accessibility of DNA without altering the DNA sequence, leading to modulation of the expression of certain genes [14]. Thus, epigenetics plays a critical role in the development of cardiovascular disease through post-translational modifications of DNA and regulation of gene expression [14]. Acetylation-mediated PTMs of protein were identified almost half a century ago and are evolutionarily conserved across species [15,16]. First, acetylation was identified in the histone protein, and then several cellular proteins were found to be acetylated [15]. Further study suggests that almost 70 percent of cellular acetylation was identified in the histone proteins [17]. Cells regulate histone protein acetylation via two classes of enzymes: lysine acyltransferases (KATs) and histone deacetylase (HDAC). Acetylation of histone protein regulates the chromatin structure and compactness and epigenetic regulation of gene expression [18]. Generally, it is found that acetylation causes the open configuration of chromatin, whereas deacetylation causes inhibition of transcription [19]. However, acetylation of cytoplasmic and mitochondrial proteins regulates the stability of proteins, enzyme activity, degradation of proteins, mitochondrial function, and cellular bioenergetics [20,21,22,23]. Acetylation of cardiac protein plays a critical role in cellular functions such as cardiac development, differentiation, sarcomere structure, and cellular metabolism [24,25,26,27,28]. Several studies previously identified the differential regulation of acetylation in cardiac proteins during disease conditions. For example, in cardiac hypertrophy, histone acetylation and deacetylation balance is dysregulated [29]. Further, studies have linked the role of HDAC with cardiac hypertrophy, and it was found that inhibition of HDAC is beneficial in reverse remodeling of the heart [30].

Here, we used an affinity-based quantitative mass spectroscopy method to identify the acetylation sites of the proteome during normoxia vs. ischemia using primary cardiomyocytes. Our study found that during ischemia, the level of total acetylation significantly reduced. Additionally, our study revealed that the acetylation of critical metabolic enzymes is altered during ischemia in cardiomyocytes.

## 2. Methods

### 2.1. Cell Culture

Neonatal Rat Primary Cardiomyocytes (NRVCs) were isolated from 1- to 2-day-old pups as described before [31]. In brief, left ventricular heart tissue was isolated from the neonatal rats and digested with trypsin (Thermo Fisher Scientific, Waltham, MA, USA) at 4 °C overnight. The next day, heart tissue was washed and digested with collagenase (Worthington Biochemical, Lakewood, NJ, USA). Cells were isolated from the digested heart tissue by differential plating. Cells were plated in the 100 mm culture disk (Genesee Scientific, San Diego, CA, USA) coated with collagen (Sigma, St Louis, MO, USA). Initially, cells were grown in MEM media (Gibco, Waltham, MA, USA) with 10% FBS (Sigma, St Louis, MO, USA) and anti-anti (ThermoFisher) and then in DMEM (ThermoFisher) media with 2% FBS and penicillin–streptomycin (ThermoFisher).

### 2.2. Ischemic Conditions

The ischemic condition was created to replicate cardiac disease conditions in the heart, where cardiomyocytes have limited access to oxygen and blood. NRVCs in DMEM (ThermoFisher) media with 2% FBS (Sigma) were replaced with DMEM (ThermoFisher) media with no glucose and incubated in the hypoxia incubator (Eppendorf, Enfield, CT, USA). The hypoxic condition was created by 5% CO_2_, 0.1% O_2_, and N_2_ gas [32]. Cells were incubated in the hypoxic condition for 12 h at 37 °C. For the control experiment, cells were incubated in normoxic conditions created by DMEM media with high glucose (ThermoFisher), 2% FBS, and 5% CO_2_ at 37 °C for 12 h.

### 2.3. Cell Harvesting and Protein Isolation

Cardiomyocytes were grown in culture plates and washed twice with cold 1X PBS buffer. Then, cells were lysed by incubating them in 0.1% RIPA buffer (150 mM NaCl, 1% IGEPAL, 50 mM Tric-HCl pH 8.0, 12 mM sodium deoxycholate, and 0.1% SDS), 1X mammalian protease inhibitor (Sigma), and 1X deacetylase inhibitors (MedChemExpress, Monmouth Junction, NJ, USA) for 10 min [33]. The cells were dislodged after incubation by scraping the plates on ice and transferring them to 1.7 mL microcentrifuge tubes. Lysed cells in the RIPA buffer were vortexed until properly mixed. The microcentrifuge tubes were centrifuged at 10,000× *g* for 10 min at 4 °C to separate cell debris, and supernatants containing proteins were collected for the experiments. Protein estimation was performed using the BCA Protein Assay Kit (ThermoFisher). Standardized dilutions were created using a protein standard to make a standard curve and determine the protein concentrations of the test samples.

### 2.4. Western Blotting

For Western blotting, protein samples were diluted with 0.1% RIPA buffer mixed with the 1X Laemmli sample and stored at −20 °C for use in further experiments. Levels of acetylation in normoxic and ischemic protein samples were detected by Western blotting as described before [34]. In brief, proteins were resolved on SDS-PAGE with a protein ladder (Proteintech, Manchester, UK) and then transferred to the PVDF membrane (Bio-Rad, Hercules, CA, USA) by electrophoresis. Proteins were transferred to the PVDF membrane by wet transfer or a trans-blot turbo transfer (Bio-Rad) according to the manufactured protocol. To block the non-specific binding of antibodies, membranes were incubated in a blocking buffer (LI-COR, Lincoln, NE, USA) for an hour at room temperature. Then, the membranes were incubated overnight at 4 °C with the primary antibodies. After washing twice with 1X PBST and once with 1X PBS, the membrane was probed with IRDye secondary antibodies (LI-COR) for 2 h at room temperature. Blots were scanned using the Odyssey scanner (LI-COR). These images were then analyzed and quantified using Image Studio (LI-COR) to compare their expression levels on the membrane. The following antibodies were used for the assay: total acetylation antibody (Cell Signaling, Middletown, DE, USA, Cat # 9814S, 1:1000), H3K9 acetylation (Abcam, Waltham, MA, USA, Cat# C5B11, 1:2000), β-actin (Proteintech, Cat# 66009-1-Ig, 1:3000), and secondary antibody goat anti-rabbit 800, goat anti-mouse 680 (LI-COR, Cat# 926-68070, 926-32211, 1:5000).

### 2.5. Protein Digestion, Affinity Purification of Acetylated Proteins, and Mass Spectrometry

A mass spectroscopy experiment was conducted to detect total protein acetylation with the help of a proteomic core (Creative Proteomics, Shirley, NY, USA) [35]. For the mass spectroscopy analysis, NRVCs were cultured in normoxic and ischemic conditions as described before. Proteins from the cultured cells were isolated using RIPA buffer. To determine the protein acetylation sites, equal amounts of proteins were pulled together from three replicates in each condition, and 2 mg pull proteins were used for the study. Proteins from each condition were diluted to 2 mL of 50 mM ammonium bicarbonate buffer. The disulfide bridges of the protein were reduced by treatment with the 10 mM Tris (2-carboxyethyl) phosphine (TCEP), and reduced cysteine residues were alkylated by 20 mM iodoacetamide (IAA). Precipitated protein was collected by centrifugation, resuspended, and digested with trypsin at an enzyme–substrate ratio of 1:200 (*w*/*w*). After digestion, TFA was added to 1% final concentration, and digested peptides were precipitated by centrifugation at 1780 g for 15 min. The peptides were dried using SpeedVac. Protein PTMs are usually in low abundance, and thus, acetylated-peptide enrichment is essential for large-scale acetylation profiling. Acetyl-peptides were enriched through acetyl lysine antibody-conjugated agarose beads (PTMBIO, Chicago, IL, USA). Agarose beads were mixed, and 40 µL of 50% bead slurry was aliquoted in a 0.6 mL tube. Beads were washed thrice with chilled PBS by centrifugation at 1000× *g* at 4 °C. The 2 mg peptides were dissolved in NETN buffer (100 mM NaCl, 1 mM EDTA, 50 mM Tris-HCl, 0.5% Nonidet P-40, pH 8.0). The peptide solutions were centrifuged at 12,000× *g* for 10 min at 4 °C to remove possible precipitates. Peptide solutions were mixed with the conjugated beads at 4 °C for 4 h with gentle shaking. Beads were washed four times with NETN buffer and twice with deionized water. Bound peptides were eluted with 1% trifluoroacetic acid. For the separation of peptides, Nanoflow UPLC (ThermoFisher Scientific), a trapping column (PepMap C18, 100 Å, 100 μm × 2 cm, 5 μm), and an analytical column (PepMap C18, 100 Å, 75 μm × 50 cm, 2 μm) were used with following conditions at a total flow rate of 250 nL/min: Mobile phase: A: 0.1% formic acid in water; B: 0.1% formic acid in 80% acetonitrile. LC linear gradient: from 2 to 8% buffer B in 3 min, from 8% to 20% buffer B in 50 min, from 20% to 40% buffer B in 26 min, and then from 40% to 90% buffer B in 4 min. The full mass spectroscopy scan was performed between 300 and 1650 *m*/*z* at the resolution 60,000 at 200 *m*/*z*, and the automatic gain control target for the full scan was set to 3 × 10^6^ The MS/MS scan was operated in Top 20 mode using the following settings: resolution 15,000 at 200 *m*/*z*; automatic gain control target 1e5; maximum injection time 19 ms; normalized collision energy at 28%; and isolation window of 1.4. The charge state exclusion was as follows: unassigned, 1, >6; dynamic exclusion 30 s. The raw MS files were analyzed using the protein database Maxquant (1.6.3.4) and searched against Rattus norvegicus. The following parameters were set during the search: carbamidomethylation (C), oxidation (M) (variables), acetyl (K) (variables), and acetyl (N-term) (variables); the enzyme specificity was set to trypsin; the maximum missed cleavages were set to 5; the precursor ion mass tolerance was set to 10 ppm; and MS/MS tolerance was 0.6 Da.

## 3. Bioinformatics Analysis

The UniProt (https://www.uniprot.org, accessed on 10 December 2021) protein database was used to annotate identified proteins’ functions in the MS experiment. For the functional enrichment analysis of the identified proteins, a GO/KEGG functional annotation tool of ShinyGO 0.77 (http://bioinformatics.sdstate.edu/go77/ accessed on 8 December 2021) was used against the Rattus Norvegicus. For motif analysis of the identified acetylation sites of the protein sequence (amino acid), a probability Logo Generator for Biological Sequence (pLogo) from SchwartZLab was used. The protein database of Rattus Norvegicus was used as a background parameter. The identified acetylated proteins were searched using the STRING v11 database for protein–protein network analysis. Subcellular localization of the identified proteins was searched using UniProt software accessed in January 2022).

## 4. Statistical Data Analysis

Statistical analysis was performed using GraphPad version 9.0 software. The results were presented as a mean ± standard deviation. For statistical significance analysis between groups, an unpaired Student *t*-test was performed. * *p* < 0.05 is considered a significant change between the groups.

## 5. Results

### 5.1. Ischemic Condition Significantly Reduces the Cellular Acetylation Level

**Figure 1:**
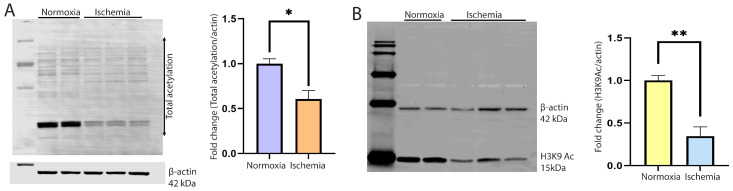
Cellular acetylation is significantly reduced during ischemia. (**A**) Western blot shows the total acetylation of the cardiomyocytes during normoxia and ischemia. NRVCs were exposed to ischemic conditions for 12 h, and a Western blot was performed with the total acetylation antibody. β-actin was used as an internal loading control. The graph shows the quantification of the Western blot (* *p* < 0.05) (**B**). The Western blot shows the acetylation of histone protein. The blot was probed with the H3K9ac antibody. The graph shows the quantification of the Western blot (** *p* < 0.05).

To monitor the changes in cellular acetylation, NRVCs were grown in no glucose and serum-free media in an ischemic condition created by reducing the oxygen level to 0.1% for 12 h at 37 °C. Control cells as a normoxia were grown in high glucose DMEM media having 2% FBS. Total proteins were isolated, and Western blots were performed with the total acetylation antibody. Our data found that the level of total acetylation in the ischemic NRVCs significantly decreased compared to the normoxic condition (Figure 1A). The published literature showed that histones are the main target of acetylation in the cells; we also detected the level of histone protein acetylation by Western blot. We used the H3K9ac antibody to detect the level of histone acetylation at lysine 9. Interestingly, we found that consistent with the total acetylation level, the level of H3K9 acetylation also significantly decreased (Figure 1B).

### 5.2. Identification of Differentially Acetylated Positions in Proteins

As our Western blot data showed that acetylation levels significantly reduced during ischemia, we are further interested in the identification of the acetylation sites of the cellular proteins. Additionally, we performed a quantitative analysis of protein acetylation to understand how ischemia modulates protein acetylation (Supplementary Figure S1). Acetylated proteins were affinity purified using acetylation affinity beads of the digested proteins. The acetylation levels of purified proteins were determined by acetyl-proteomics using mass spectrometry. Based on the mass spectroscopy data, the differentially acetylated proteins in ischemic conditions were identified (Table 1). In this experiment, a total of 179 acetylation sites were identified (Table 1). The acetylation quantification showed that acetylation at 26 sites was upregulated (UP), as determined by a fold change greater than 1.5. There are 121 sites that are downregulated (DOWN), determined by a fold change of less than 1/1.5. There are 30 sites where it was unable to be determined if acetylation was upregulated or downregulated, labeled no change (NC). Additionally, we determined the function of identified proteins using the UniProt (https://www.uniprot.org) database (Table 2).

### 5.3. Sequence Motifs of Lysine Acetylation Sites

**Figure 2:**
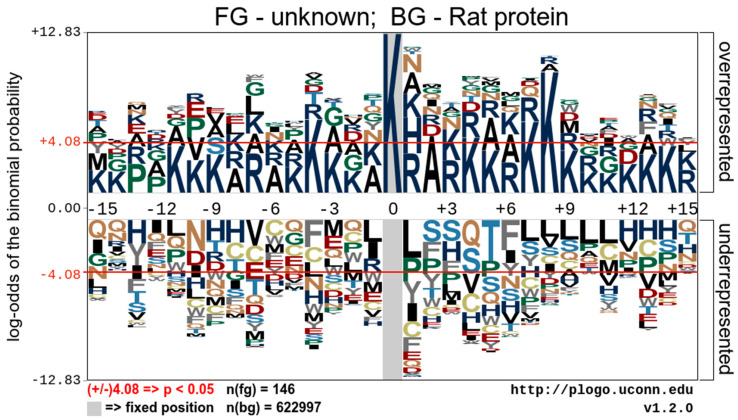
Analysis of the acetylation motifs of acetylated peptides identified by mass spectroscopy. A representative image shows the overrepresented and underrepresented amino acid residues surrounding acetylated lysine.

To understand the regulation of protein acetylation and identify the occupancy frequency of the surrounding amino acids of the identified proteins’ acetylation sites, we visualized the Kac protein motifs using the pLogo bioinformatic tool. The data show an overrepresentation of lysine at positions −4, 4, 7, and 8 relative to the acetylated lysine. It also contains an overrepresentation of alanine at position 2 relative to the acetylated lysine. There were no statistically significant underrepresented sequence motifs (Figure 2).

### 5.4. Functional Enrichment Analysis of the Acetylated Proteins

**Figure 3:**
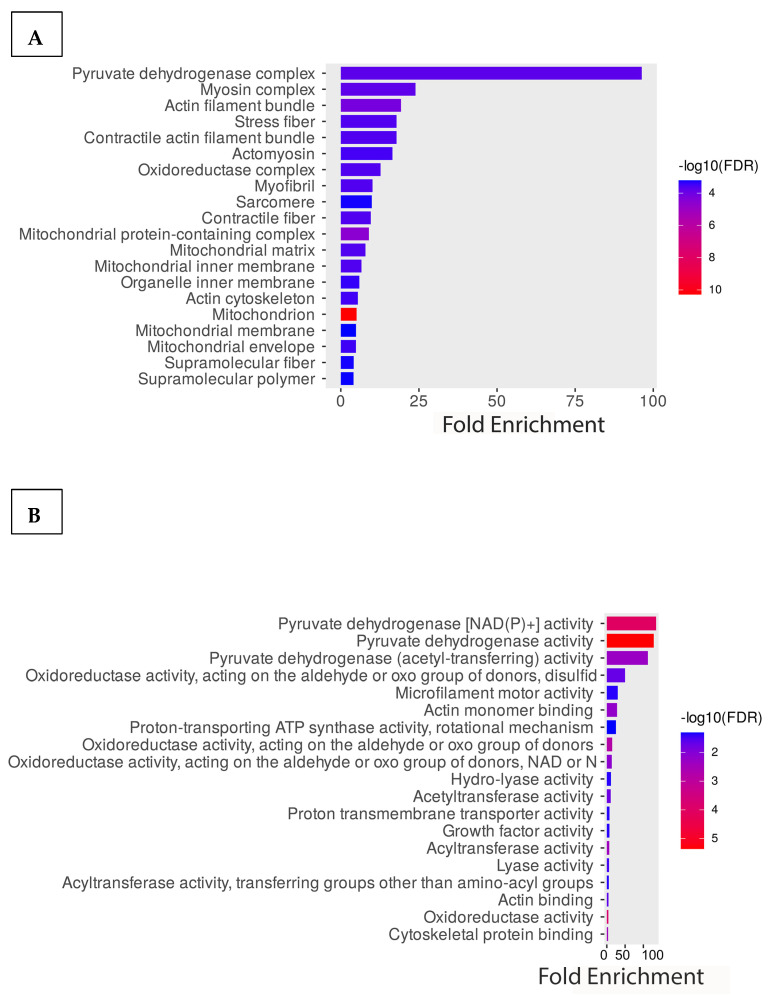

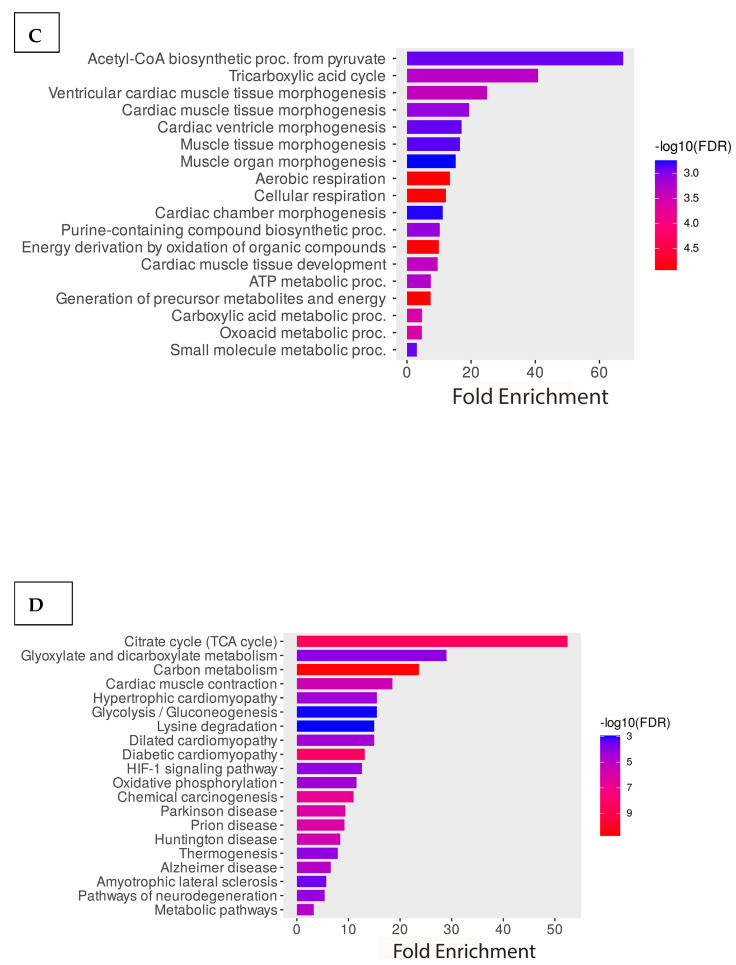
Functional enrichment analysis of the acetylated proteins. GO enrichment analysis of the proteins is based on (**A**) cellular components, (**B**) molecular functions, and (**C**) biological processes. (**D**) KEGG pathway enrichment analysis of the proteins.

To better understand the acetylome of cardiomyocytes, we performed GO enrichment analysis of all identified proteins based on biological processes, molecular functions, and cellular components. The graph below illustrates the percentage of genes out of all the differentially associated proteins that are associated with a particular cellular component compared to the baseline percentage of genes in the background. The result indicated that the cellular component containing the highest comparative number of differentially acetylated genes is the pyruvate dehydrogenase complex (Figure 3A). Additionally, cellular contractile proteins like myosin, actin, and stress fibers are enriched. Consistent with the cellular component result, the molecular function is also enriched with the metabolic enzymes involved in pyruvate dehydrogenase activity. Other enriched proteins belong to the category of oxidoreductase and motor activity of the cells (Figure 3B). Further enrichment analysis of the biological processes shows that most enriched proteins are involved in acetyl-CoA biosynthesis from pyruvate, the tricarboxylic acid cycle, and muscle tissue morphogenesis. Based on the significance, the highest enriched groups in the biological process are involved in cellular respiration (Figure 3C). Additionally, we performed an enrichment analysis of the identified protein using the KEGG pathways. Interestingly, consistent with the GO molecular functional analysis, highly enriched proteins are involved with cellular metabolic pathways such as the TCA, glyoxylate metabolism, carbon metabolism, pyruvate metabolism, and cardiac muscle contraction (Figure 3D).

We also used UniProt software to determine the identified protein’s subcellular localization in the cell. Our analysis showed that acetylated proteins are localized all over the cell, including the cytoplasm, cytoskeleton, endoplasmic reticulum, extracellular region, cell membrane, mitochondria, nucleus, ribosome, and peroxisome. However, most proteins are localized in mitochondria, the nucleus, and the cytoplasm (Supplementary Figure S2).

### 5.5. String Protein Web

**Figure 4:**
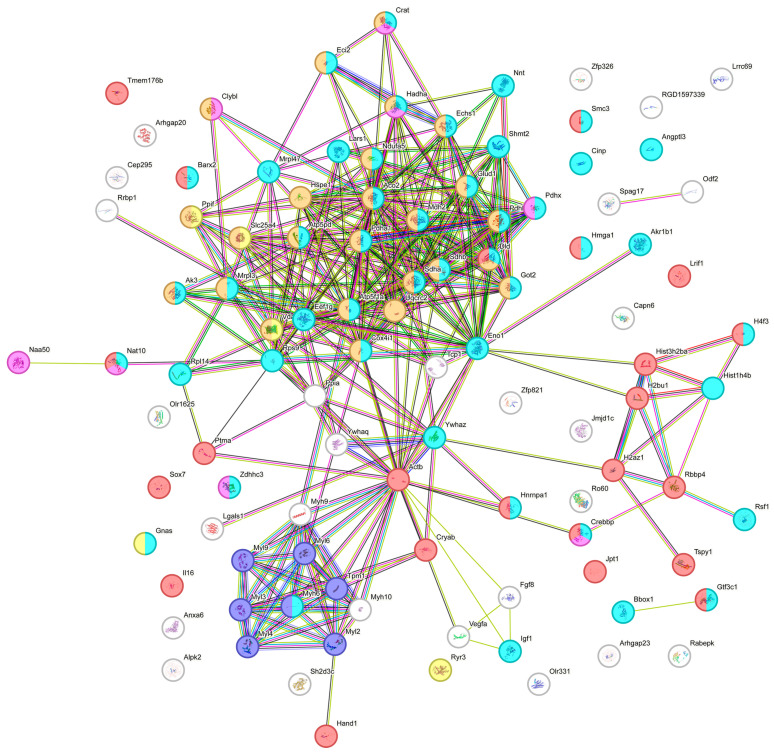
STRING protein web grouping showing the primary functions of differentially acetylated proteins.

A STRING protein web highlights the primary functions of acetylated proteins, indicating the groupings of physiological processes affected by lysine PTM. Many differentially acetylated proteins are connected with cellular metabolism, and this cluster of proteins was labeled cyan. The mitochondrial proteins labeled as brown are heavily involved in the transport of electrons and ATP synthesis, and they are critical in the process of oxidative phosphorylation. Ischemic conditions affecting these processes result in mitochondrial dysfunction and the development of disease conditions such as cardiovascular disease, stroke, etc. The cluster of proteins labeled in purple, such as the myosin regulatory light polypeptides 2, 3, 4, 6, and 9, plays a primary role in striated muscle contraction by regulating the movement of myosin head molecules for cross-bridge formation. These proteins are connected and primarily affect myosin 6, tropomyosin alpha-1 chain, and actin. Actin has key functions in cell motility and contraction in the cytoplasmic cytoskeleton. In addition, G- and F-actin regulate gene transcription, motility, and the repair of damaged DNA by localizing in the nucleus. Another grouping of deferentially acetylated nuclear proteins included histone cluster 1 H1 family member d, Histone H4-like protein, Histone H2A.Z variant, and Histone 2B. These proteins labeled red provide structural support for the chromatin and are also connected to RB-binding protein 4 and remodeling and spacing factor 1, which modulate chromatin organization and remodeling (Figure 4). The calcium and muscle contraction regulatory proteins were labeled as yellow. We also identified proteins involved in acyltransferase activity, such as Nat10 and Crebbp, which were labeled pink.

The functional importance of identified acetylated proteins in the heart is as follows:

Recently, the post-translational modifications of cellular proteins have received special attention due to the multiple roles they play in regulating cellular functions. Acetylation of cardiac proteins regulates several cellular functions, including gene expression, mitochondrial energy homeostasis, calcium homeostasis and calcium sequestration, cellular metabolism, cellular protein degradation and stability, signal transduction, and cardiac contractility [24,36]. Mitochondrial function: Our study identified several mitochondrial proteins that are differentially regulated during ischemia (Table 1). Studies suggest that hyperacetylation of mitochondrial proteins reduces cardiac energetics and promotes the development of oxidative stress and pathophysiological conditions. One of the critical enzymes enriched in the proteomic study is the pyruvate dehydrogenase complex (PDH). Its acetylation during ischemic conditions significantly increased. PDH is an important enzyme that connects glycolysis to the tricarboxylic acid (TCA) cycle pathway through the conversion of pyruvate to acetyl-CoA. Previous studies showed that increased acetylation compromises the enzyme activity of PDH and decreases the cellular acetyl-CoA level [37,38]. ATP5f1c is another mitochondrial protein found to be hyperacetylated in our study. ATP5f1c is a mitochondrial ATP synthase that generates ATP during oxidative phosphorylation. Studies suggest that hyperacetylation of this enzyme interferes with enzyme activity and induces metabolic dysfunction and senescence in the cardiac cells [39].

Gene regulation and transcription: Further, acetylation of nuclear proteins plays an important role in chromatin structure and gene expression, stress response of cells, and development of cardiac disease. Our study identified several acetylated nuclear proteins, including histones (Table 1). We found that acetylation of histones significantly decreased during ischemia. Also, we have identified a cytidine acetyltransferase Nat10, which was hyperacetylated during ischemia. Nat10 plays a critical role in the regulation of Ac4C-mediated epigenetic regulation of mRNAs [40,41]. A study with a mouse model found that knockdown of Nat10 induces cardiomyocyte apoptosis and heart failure [42]. Further, it was found that Ac4C-mediated modification of mRNA enhances the stability of cellular protein BCL-XL, which promotes myocardial infarction-induced cardiac fibrosis development [43]. Furthermore, it was found that Nat10 can undergo autoacetylation, which is important for its activity [44]. Additionally, Nat10 plays a critical role during cellular energy stress through the regulation of cellular rRNA synthesis and autophagy to maintain cellular energy supply [45].

Metabolism: Growing evidence also suggests that acetylation of a cellular protein is critical for cardiac metabolism and energy balance. Metabolic enzymes involved in fatty acid oxidation and glucose metabolism are known to be acetylated. Our study found that several metabolic proteins involved in glycolysis, the TCA cycle, and fatty acid oxidation were differentially acetylated (Table 1). One of the metabolic enzymes found to be acetylated in ischemic conditions is insulin-like growth factor (IGF-1). IGF-1 plays a critical role in cardiovascular function through the regulation of cellular metabolism, development, cellular contractility, and heart function by regulating insulin levels, insulin sensitivity, and glucose metabolism [46,47]. Decreased expression of IGF-1 during myocardial infarction was linked with a worse prognosis [48]. Interestingly, it was found that the metabolic status of the heart can regulate the acetylation of IGF-1 [46]. Further, it was evident that IGF-1 regulates metabolic enzymes ENO2 and SIRT1 via their acetylation and cellular transcription by histone 3 and histone 4 acetylation [49].

Sarcomeric proteins and contractility: Acetylation also plays a critical role in the regulation of cellular contractility through calcium homeostasis [50,51]. In our study, we found that several sarcomeric proteins such as myosin, actin, and tropomyosin become acetylated. Studies suggested that acetylation of sarcomeric proteins plays a significant role in the stiffness of the sarcomere [52]. It was found that acetylation of sarcomeric protein titin causes less muscle stiffness, and HDAC6 can cause more titin stiffness, which can impact heart function [53]. Additionally, it was found that acetylation of myosin modulates its enzyme activity as well as motor function. We also detected the acetylation of the RyR3 protein at the divergent region 1 (DR1). DR1 region plays a critical role in the regulation of calcium homeostasis in the sarcoplasmic reticulum and actomyosin movement of the sarcomere [54,55]. We also detected actin acetylation in the cardiomyocytes. It was found that lysine acetylation of the actin protein can decrease the tropomyosin-mediated regulation of actomyosin activity [56].

Stress response: Acetylation also plays an important role in the stress response, protein folding, stability, and degradation of cardiac cells. One of the stress-induced proteins, TCP, was found to be acetylated in the ischemic cells. TCP plays a critical role in folding several cellular proteins, including myosin, actin, and tubulin [57]. Knockdown of the TCP protein can impact heart function [57]. Additionally, it was demonstrated that acetylation of TCP significantly increased during heat shock [58].

**Figure 5:**
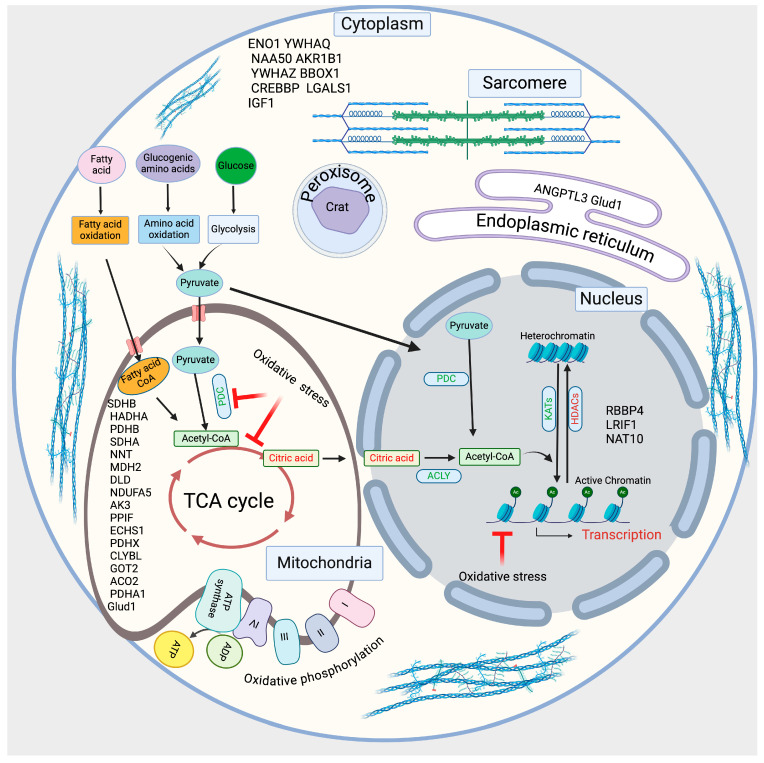
The schematic diagram shows the identified acetylated proteins involved in regulating cellular metabolism and acetylation.

Additionally, we drew a schematic diagram based on the acetylated proteins identified in our study, which are involved in cellular metabolism, acetylation of cellular proteins, mitochondrial function, gene regulation, and sarcomere functions (Figure 5).

## 6. Discussion

Acetylation of lysine is a conserved post-translation protein modification, which plays many important roles, including those involved in gene transcription, cellular metabolism, and cell survival via altering the function of proteins [42,43,44,45,46,59,60,61,62,63,64,65]. The current study aims to identify proteins that have been differentially acetylated due to ischemic conditions and analyze what biological pathways are the most impacted. The results show that 179 proteins were differentially modified due to post-translational modification by acetylation during ischemia. Bioinformatics analysis indicates that these acetylated changes are found to be localized in different subcellular organelles like mitochondria, nucleus, cytoplasm, etc. Protein enrichment analysis revealed that post-translationally modified proteins are involved in various cellular functions, such as protein synthesis, epigenetic modification, and metabolism. Our study identified major biological pathways that may be impacted by ischemic conditions, such as the acetyl-coA biosynthetic process from pyruvate, the tricarboxylic acid cycle, and ventricular cardiac muscle tissue morphogenesis. The cellular processes most impacted (pyruvate dehydrogenase complex) and molecular functions most impacted (pyruvate dehydrogenase NADP+ activity) were also identified. The pyruvate dehydrogenase complex catalyzes the oxidative decarboxylation of pyruvate to form acetyl CoA [66]. It plays a major role in aerobic respiration as the essential link between glycolysis (anaerobic metabolism) and the tricarboxylic acid cycle [66].

Dysregulated acetylation and hyperactivity of lysine deacetylase (KDAC) enzymes are involved in cardiac dysfunction, and many other proteins may have similar effects. It is known that ischemic/reperfusion (I/R)-induced tissue injury can cause deacetylation through HDAC. The HDAC class of proteins removes acetyl groups from histones and non-histone proteins, which regulates the chromatin structure that turns off gene expression [67]. Dysregulated acetylation is identified in Figure 1 of this study, as total acetylation is significantly decreased in ischemic conditions. HDAC inhibitors have been reported to show beneficial outcomes for cardiac arrhythmia, cardiac fibrosis, cardiac hypertrophy, and myocardial infarction. Trichostatin A is a pan-HDAC inhibitor, which inhibits Class I and Class II HDACs and has been shown to decrease the gene expression levels of hypoxia-inducible factor-1 (HIF-1) protein and the genes that it targets [67]. This signaling can promote cell survival, reduce vascular permeability, and ultimately reduce myocardial injury [67]. H3K9 acetylation plays a critical role in the regulation of transcription initiation. Studies suggest that H3K9 acetylation acts as a substrate of the super elongation complex (SEC) on chromatin. This complex formation promoted the pol II pause release and progression of the transcription cycle. In our study, H3K9 acetylation significantly decreased in ischemic conditions (Figure 1).

## 7. Future Perspectives

Increasing evidence shows that acetylation-mediated post-translational modification of cardiac proteins plays an important role in the cellular metabolism and development of CVDs. In the past, advancements have been made to identify the acetylation of proteins and their role in CVD. Our acetylome study provides a comprehensive understanding of the acetylation of proteins and its regulation during ischemia. Organizing and categorizing differentially acetylated proteins can be useful in identifying new potential targets for epigenetic-based therapy of ischemic heart disease. Combining the use of epidrugs with conventional therapy has been newly identified as beneficial in the treatment of patients with heart failure [68]. Currently, there are some drugs on the market that take advantage of the reversible nature of epigenetic-sensitive changes, such as vorinostat (Zolinza), belinostat (Beleodaq), romidepsin (Istodax), and panobinostat (Farydak), which all function as histone deacetylase inhibitors (HDACis) [69]. In our study, we found that many mitochondrial proteins become acetylated during ischemia. Additionally, to our knowledge, we identified the acetylation of RyR3 for the first time. However, the role of acetylation in the DR1 in the regulation of cardiac calcium homeostasis and contractility is not clear. Therefore, more studies are needed to investigate the regulation of organ-specific acetylation, specifically the mitochondrial proteins, to modulate cardiac energy homeostasis.

## 8. Conclusions

PTMs play a critical role in the regulation of cardiac function, metabolism, and epigenetic regulation through the regulation of enzyme activity, its stability, and its localization. Our study identified several known and unknown acetylation sites in several cardiomyocyte proteins. Further studies are needed to explore the role of each identified acetylation of proteins and its connection to the cellular protein function, substrate binding, and localization. Additionally, by modulating the protein acetylation, it may be possible to alter cardiac energy status during disease conditions and improve CVDs. This study was limited due to time constraints and the availability of resources. However, given more time, specific proteins can be further explored to see how the changes in acetylation impact the protein’s stability and enzymatic activity and can provide a new drug target for CVDs.

## Figures and Tables

**Table 1:** List of acetylated proteins identified in mass spectroscopy.

	Protein Names	Acetylated Positions	Protein	Acetylation
1	ATP synthase subunit alpha; mitochondrial	259; 261	F1LP05	UP
2	14-3-3 protein zeta/delta	60; 78; 51; 50	A0A0G2JV65	UP
3	Hyaluronan-mediated motility receptor	80	A0A0G2JXY1	UP
4	Hyaluronan-mediated motility receptor	83	A0A0G2JXY1	UP
5	Ro60, Y RNA-binding protein	489	D3ZRN5	DOWN
6	CREB-binding protein	1584	F1M9G7	DOWN
7	CREB-binding protein	1587	F1M9G7	DOWN
8	CREB-binding protein	1588	F1M9G7	DOWN
9	Aldose reductase	263	P07943	UP
10	40S ribosomal protein S9	155	P29314	NC
11	14-3-3 protein theta	49	P68255	UP
12	Rab9 effector protein with kelch motifs	2	Q4V8F4	UP
13	Galectin-1	29	P11762	UP
14	Hematological and neurological expressed 1 protein	143	Q6AXU6	UP
15	Gamma-butyrobetaine dioxygenase	158	A0A0G2K461	UP
16	Tropomyosin alpha-1 chain	260	A0A0G2K7F7	UP
17	Sperm-associated antigen 17	142	M0R8D9	DOWN
18	Sperm-associated antigen 17	147	M0R8D9	DOWN
19	Calpain-6	616	G3V6M4	NC
20	Myosin, heavy polypeptide 9, nonmuscle	1352	G3V6P7	UP
21	Myosin-6; Myosin-7	1833; 1831	G3V885	UP
22	Myosin-6	1643	G3V885	NC
23	Myosin-6; Myosin-7	1487; 1485	G3V885	UP
24	Myosin-6; Myosin-7	1501; 1499	G3V885	UP
25	Myosin-6; Myosin-7	1365; 1363	G3V885	UP
26	Myosin light chain 3	38	P16409	UP
27	Myosin light chain 4	123; 123	P17209	UP
28	Centrosomal protein of 295 kDa	1478	A0A0G2K417	NC
29	Outer dense fiber protein 2	617	G3V7X0	UP
30	Outer dense fiber protein 2	620	G3V7X0	UP
31	T-complex protein 1 subunit alpha	400	P28480	UP
32	Serine hydroxymethyltransferase	409	Q5U3Z7	UP
33	Myosin-10	1055	G3V9Y1	UP
34	Myosin-10	1057	G3V9Y1	UP
35	Myosin regulatory light polypeptide 9	7	B0BMS8	UP
36	Myosin regulatory light polypeptide 9	12	B0BMS8	UP
37	Myosin regulatory light polypeptide 9	13	B0BMS8	UP
38	Myosin light polypeptide 6	81	A0A0G2K6J5	NC
39	Myosin-4; Myosin-6; Myosin-7	1330; 1328; 1326	G3V885	UP
40	Alpha-enolase	5	M0R5J4	UP
41	60S ribosomal protein L19	153	M0RDT4	UP
42	60S ribosomal protein L19	163	M0RDT4	UP
43	60S ribosomal protein L29	45	A0A0G2QC62	UP
44	60S ribosomal protein L30	46	A0A0G2QC62	UP
45	60S ribosomal protein L31	49	A0A0G2QC62	UP
46	Leucyl-tRNA synthetase	143	Q5PPJ6	DOWN
47	Leucyl-tRNA synthetase	145	Q5PPJ6	DOWN
48	Leucyl-tRNA synthetase	146	Q5PPJ6	DOWN
49	Leucyl-tRNA synthetase	148	Q5PPJ6	DOWN
50	Ribosome-binding protein 1	772	F1M853	UP
51	Glutamate dehydrogenase 1, mitochondrial	480	P10860	UP
52	Elongation factor 1-gamma	147	Q68FR6	UP
53	Angiopoietin-like 3	425	F7FHP0	DOWN
54	Uncharacterized protein	135	A0A0G2JT80	DOWN
55	Insulin-like growth factor I	126	A0A0G2JX40	UP
56	Insulin-like growth factor I	127	A0A0G2JX40	UP
57	Vascular endothelial growth factor A	327	A0A0H2UHY5	DOWN
58	Vascular endothelial growth factor A	330	A0A0H2UHY5	DOWN
59	Fibroblast growth factor	52	Q76LI5	UP
60	Fibroblast growth factor	60	Q76LI5	UP
61	Pro-interleukin-16	487	D4A4I9	NC
62	Alpha-crystallin B chain	103	P23928	UP
63	RCG48016, isoform CRA_c	67	D4AAM1	UP
64	RCG48016, isoform CRA_c	68	D4AAM1	UP
65	Palmitoyltransferase	264	Q2TGK3	NC
66	Annexin	370	Q6IMZ3	UP
67	ADP/ATP translocase 1	23	Q6P9Y4	UP
68	ADP/ATP translocase 1	96	Q6P9Y4	UP
69	Pyruvate dehydrogenase E1 component subunit alpha; mitochondrial	243	F7FKI5	UP
70	Dihydrolipoamide acetyltransferase component of pyruvate dehydrogenase complex	195	A0A0G2JZH8	UP
71	Voltage-dependent anion-selective channel protein 3	63	A0A0G2JSR0	UP
72	39S ribosomal protein L3, mitochondrial	213	G3V7P3	DOWN
73	Similar to orotein C6orf203	149	M0R7R2	UP
74	Malate dehydrogenase, mitochondrial	185	P04636	UP
75	Malate dehydrogenase, mitochondrial	301	P04636	UP
76	Malate dehydrogenase, mitochondrial	157	P04636	UP
77	Cytochrome c oxidase subunit 4 isoform 1, mitochondrial	29	P10888	UP
78	Enoyl-CoA hydratase, mitochondrial	101	P14604	UP
79	Succinate dehydrogenase [ubiquinone] iron–sulfur subunit, mitochondrial	64	P21913	NC
80	10 kDa heat shock protein, mitochondrial	80	P26772	UP
81	Peptidyl-prolyl cis–trans isomerase F, mitochondrial	72	P29117	UP
82	GTP: AMP phosphotransferase AK3, mitochondrial	34	Q6P2A5	UP
83	ATP synthase subunit d, mitochondrial	71	P31399	UP
84	Cytochrome b-c1 complex subunit 2, mitochondrial	91	P32551	UP
85	Mitochondrial ribosomal protein L47	146	Q3B8R7	UP
86	Proton-translocating NAD(P)(+) transhydrogenase	403	Q5BJZ3	UP
87	Proton-translocating NAD(P)(+) transhydrogenase	768	Q5BJZ3	UP
88	Citrate lyase subunit beta-like protein, mitochondrial	55	Q5I0K3	UP
89	NADH dehydrogenase [ubiquinone] 1 alpha subcomplex subunit 5	40	Q63362	UP
90	Trifunctional enzyme subunit alpha, mitochondrial	60	Q64428	UP
91	Trifunctional enzyme subunit alpha, mitochondrial	46	Q64428	UP
92	Succinate dehydrogenase [ubiquinone] flavoprotein subunit, mitochondrial	242	Q920L2	UP
93	Aconitate hydratase, mitochondrial	50	Q9ER34	UP
94	Pyruvate dehydrogenase E1 component subunit beta, mitochondrial	132	A0A0G2KAM3	UP
95	Carnitine O-acetyltransferase	390	A0A0H2UI21	UP
96	Enoyl-CoA delta isomerase 2, mitochondrial	90	Q5XIC0	NC
97	Myosin regulatory light chain 2, ventricular/cardiac muscle isoform	165	P08733	UP
98	Myosin regulatory light chain 2, ventricular/cardiac muscle isoform	62	P08733	UP
99	Zinc finger protein 326	287	M0R440	UP
100	Histone domain-containing protein	16	A0A0G2K7R1	UP
101	Histone domain-containing protein	17	A0A0G2K7R1	UP
102	Histone domain-containing protein	21	A0A0G2K7R1	DOWN
103	Histone domain-containing protein	24	A0A0G2K7R1	UP
104	Histone domain-containing protein	13	A0A0G2K7R1	NC
105	Histone domain-containing protein	12	A0A0G2K7R1	NC
106	Histone H2A; Histone H2A.Z	7	A0A0A0MXW3	UP
107	Histone H2A; Histone H2A.Z	11	A0A0A0MXW3	UP
108	RB-binding protein 4, chromatin-remodeling factor	4	B5DFB2	UP
109	Remodeling and spacing factor 1	1065	D3ZGQ8	UP
110	Histone H3; Histone H3.1; Histone H3.3	80	M0RBX6	UP
111	Histone H3; Histone H3.1; Histone H3.3	19	M0RBX6	UP
112	Histone H3; Histone H3.1; Histone H3.3	24	M0RBX6	UP
113	Histone H3; Histone H3.1; Histone H3.3	57	M0RBX6	UP
114	Structural maintenance of chromosomes protein	105	F1LQB2	UP
115	Structural maintenance of chromosomes protein	106	F1LQB2	UP
116	BARX homeobox 2	16	D4A7E7	UP
117	RNA cytidine acetyltransferase	8	D4AEB4	UP
118	Jumonji domain-containing 1C	345	F1LMK8	UP
119	H1.3 linker histone, cluster member	187	M0R7B4	UP
120	H1.3 linker histone, cluster member	191	M0R7B4	UP
121	H1.3 linker histone, cluster member	193	M0R7B4	UP
122	Prothymosin alpha	103	P06302	NC
123	Heart- and neural crest derivative-expressed protein 1	101	P97832	UP
124	High mobility group protein HMG-I/HMG-Y	7	Q8K585	UP
125	Transmembrane protein 176B	258	Q925D4	NC
126	Histone H2B	17	A0A0G2JXE0	NC
127	Histone H2B	21	A0A0G2JXE0	UP
128	Histone H2B	13	A0A0G2JXE0	NC
129	Histone H2B	16	A0A0G2JXE0	NC
130	Histone H2B; Histone H2B type 1; Histone H2B type 1-A	109; 109; 109	M0RBQ5	UP
131	Histone H2B	12	A0A0G2JXE0	
132	Histone H2B; Histone H2B type 1	16	M0RBQ5	NC
133	Histone H2B; Histone H2B type 1	17	M0RBQ5	NC
134	Histone H2B; Histone H2B type 1	21	M0RBQ5	NC
135	Histone H2B; Histone H2B type 1	24	M0RBQ5	NC
136	Histone H2B; Histone H2B type 1	13	M0RBQ5	NC
137	Histone H2B	12	M0RBQ5	NC
138	Zinc finger protein 821	305	D3ZEI3	UP
139	General transcription factor 3C polypeptide 1	1220	F1LNV7	NC
140	General transcription factor 3C polypeptide 1	1222	F1LNV7	NC
141	Testis-specific Y-encoded protein 1	176	Q9R1M3	NC
142	Heterogeneous nuclear ribonucleoprotein A1	3	Q6P6G9	UP
143	Actin, cytoplasmic 1	61; 61; 63	P60711	UP
144	Histone H4; osteogenic growth peptide	9	P62804	DOWN
145	Histone H4; osteogenic growth peptide	13	P62804	DOWN
146	Histone H4; osteogenic growth peptide	17	P62804	DOWN
147	Histone H4; osteogenic growth peptide	6	P62804	DOWN
148	Dihydrolipoyl dehydrogenase, mitochondrial	334	Q6P6R2	UP
149	SRY-box containing gene 7 (predicted)	119	D3ZTE1	UP
150	SRY-box containing gene 7 (predicted)	120	D3ZTE1	UP
151	SRY-box containing gene 7 (predicted)	123	D3ZTE1	UP
152	Acyl-coenzyme A amino acid N-acyltransferase 1	309	A0A0G2K2H6	UP
153	Guanine nucleotide-binding protein G(s) subunit alpha isoforms XLas	756	A0A0G2JWA1	
154	Olfactory receptor	295	D3ZL36	UP
155	Olfactory receptor	302	D3ZL36	UP
156	Olfactory receptor	295	D3ZSI0	UP
157	Olfactory receptor	296	D3ZSI0	UP
158	ATP synthase subunit alpha, mitochondrial	161	F1LP05	UP
159	Alpha-kinase 2	1728	F1LTG2	UP
160	Aspartate aminotransferase, mitochondrial	159	P00507	UP
161	Aspartate aminotransferase, mitochondrial	296	P00507	UP
162	N-alpha-acetyltransferase 50-like	33	M0RBI3	UP
163	60S ribosomal protein L14	162	F1LSW7	UP
164	60S ribosomal protein L15	163	F1LSW7	UP
165	Ryanodine receptor 3	4351	F1LPJ2	UP
166	Ryanodine receptor 4	4358	F1LPJ2	UP
167	SH2 domain-containing 3C	45	B0BN10	UP
168	Leucine-rich repeat-containing 69	14	D3Z998	DOWN
169	Leucine-rich repeat-containing 70	17	D3Z998	DOWN
170	Leucine-rich repeat-containing 71	24	D3Z998	DOWN
171	Ligand-dependent nuclear receptor-interacting factor 1	90	B0BNF2	NC
172	Ligand-dependent nuclear receptor-interacting factor 1	91	B0BNF2	NC
173	Peptidyl-prolyl cis–trans isomerase A	79	A0A0G2K1P0	UP
174	Peptidyl-prolyl cis–trans isomerase A	34	A0A0G2K1P0	NC
175	Cyclin-dependent kinase 2-interacting protein	108	B0BNM8	DOWN
176	Rho GTPase-activating protein 23	684	F1M2D4	DOWN
177	Rho GTPase-activating protein 24	685	F1M2D4	DOWN
178	Rho GTPase-activating protein 20	1052	Q6REY9	NC
179	Rho GTPase-activating protein 20	1054	Q6REY9	NC

**Table 2:** Functional annotations of genes.

	Gene Symbol	Functional Annotations
1	Sdha	Succinate dehydrogenase is a mitochondrial flavoprotein (FP), a subunit of succinate dehydrogenase (SDH), which participates in the electron transport chain process of mitochondria.
2	Myl9	Myosin regulatory light polypeptide 9 plays an important role in the regulation of smooth muscle and nonmuscle cell contractility through a phosphorylation-dependent manner. Additionally, it plays an important role in other cellular processes like cell division, cell movement, and receptor capping.
3	Ndufa5	NADH dehydrogenase 1 alpha subcomplex subunit 5 constitutes the NADH dehydrogenase Complex I and helps in the transfer of electrons from NADH to the respiratory chain.
4	Atp5f1a	ATP synthase subunit alpha is a mitochondrial membrane protein. It generates ATP using the proton gradient of the membrane.
5	Eno1	Alpha-enolase is a glycolytic enzyme that produces phosphoenolpyruvate from 2 phosphoglycerate. It regulates several cellular functions, including cellular growth, hypoxia, biochemical reactions, allergic reactions, etc.
6	Ryr3	Ryanodine receptor 3 regulates the cellular calcium and contractility of cells.
7	Zfp821	Zinc finger protein 821 is a nuclear DNA-binding protein.
8	RGD1597339	The function of RCG48016, isoform CRAc, is not known.
9	Igf1	Insulin-like growth factor I functions similarly to insulin but shows higher growth-promoting activity. It may participate in the transport of glucose and glycogen synthesis.
10	Alpk2	Alpha-kinase 2 plays an important role in DNA repair and cellular apoptosis.
11	Clybl	Citramalyl-CoA lyase regulates the vitamin B12 metabolism. It also regulates the cellular detoxification process through detoxifying itaconate. Additionally, it also catalyzes the malate and beta-methyl malate synthesis in vitro.
12	Fgf8	Fibroblast growth factor is a heparin-binding protein. It plays an important role in embryonic development, cell proliferation, cell differentiation, and cell migration. Additionally, it is required for the development of the gonadotropin-releasing hormone neuronal system.
13	Odf2	Outer dense fiber protein 2 is an important component of the mammalian sperm tail and regulates sperm motility. It also functions as a general scaffold protein on the distal/subdistal appendages of the mother centrioles.
14	Myh10	Myosin-10 regulates the stabilization of type I collagen mRNAs by interaction with the LARP6. It plays an important role in cytoskeleton reorganization and regulates lamellipodial extension, cell division, and cell shape.
15	Pdha1	Pyruvate dehydrogenase E1 component subunit alpha is a mitochondrial protein and regulates cellular glycolytic and TCA cycles.
16	Zfp326	Zinc finger protein 326 belongs to the AKAP95 family.
17	Actb	Actin forms cross-linked networks in the cell’s cytoplasm. Its monomeric (G-actin) and polymeric (F-actin) forms regulate cell motility and contraction in the cytoplasmic cytoskeleton. Nuclear actin regulates gene transcription, motility, and repair of damaged DNA.
18	Olr1625	This is an olfactory receptor and belongs to the G-protein-coupled receptor.
19	Barx2	BARX homeobox 2 is a DNA-binding transcriptional factor. It regulates the expression of neural adhesion molecules such as L1 or Ng-CAM during the embryonic development of the nervous system.
20	Hnrnpa1	Heterogeneous nuclear ribonucleoprotein A1 regulates the packaging of pre-mRNA into hnRNP particles. It also regulates the transport and splicing of mRNA.
21	Bbox1	Gamma-butyrobetaine dioxygenase regulates the L-carnitine formation from gamma- butyrobetaine.
22	H4f3	This is histone cluster 1 H1 family member d.
23	Hand1	Heart- and neural crest derivative-expressed protein 1 is a transcriptional factor, regulates cell differentiation, and is involved in cardiac morphogenesis. It also acts as a transcriptional repressor of the SOX15 gene, involved in cardiac oncogenesis.
24	Jmjd1c	Jumonji domain-containing 1C histone demethylase plays an important role in histone code via the demethylation of Lys-9 of histone H3.
25	Hmga1	High mobility group protein HMG-I/HMG-Y preferably binds with the A- and T-rich double-stranded DNA. These proteins could function in nucleosome phasing and in the 3′-end processing of mRNA transcripts. Additionally, it regulates the transcription of genes belonging to the HMGA family.
26	Ppia	Peptidyl-prolyl cis–trans isomerase is involved in several cellular functions including protein folding, cellular apoptosis and cell death, platelet activation and aggregation, ROS production, and clearance of protein aggregates.
27	Olr331	Olfactory receptor belongs to G-protein-coupled receptors. This helps in the initiation of neuronal response that helps in the perception of a smell.
28	H2bu1	Histone H2B belongs to the histone H2B family.
29	Sdhb	Succinate dehydrogenase iron–sulfur subunit is a mitochondrial protein involved in mitochondrial electron transport and energy production. It also regulates the oxygen-related gene transcription through the production of succinate. Succinate is an oxygen sensor that stabilizes the hypoxia-inducible factor 1 (HIF1).
30	Gnas	Neuroendocrine secretory protein 55 belongs to the NESP55 family. This protein is involved in cellular signal transduction through G-protein-coupled receptors.
31	Ptma	Prothymosin alpha is an N-terminally processed protein found to be localized in the nucleus, cytoplasm, and extracellular environments. It regulates several cellular processes, including chromatin remodeling, transcription regulation, and inhibition of cellular apoptosis by blocking apoptosome formation.
32	Capn6	Calpain-6 is a microtubule-stabilizing protein that could regulate cytoskeletal organization and microtubule dynamics. It may also control lamellipodial formation and cell mobility, but it does not seem to have any protease activity.
33	Zdhhc3	Palmitoyltransferase helps in palmitoyltransferase activity. Zdhhc3 protein was found to be localized in the Golgi apparatus, and it is involved in several cellular functions, including TRAIL-activated apoptosis and protein localization in the membrane.
34	Arhgap23	Rho GTPase-activating protein 23 is a small GTPase and is involved in signal transduction through transmembrane receptors. Arhgap23 has an inactive GDP-bound form and an active GTP-bound form. It regulates the activity of RHO family proteins by stimulating their hydrolysis of GTP.
35	Hist1h4b	This is Histone H4-like.
36	Crebbp	Crebbp regulates histone acetylation by its histone lysine acetyltransferase activity and regulates cellular gene expression in association with the CREB. It plays a critical role in cellular growth and division.
37	Ro60	Ro60 is an RNA-binding protein that binds with the misfolded non-coding RNAs. It regulates the folding and degradation of non-coding RNAs.
38	Vegfa	Vascular endothelial growth factor A regulates several cellular functions, including angiogenesis, vasculogenesis, endothelial cell growth, and vascular permeability during lactation.
39	Lars1	Lars1 functions as a cytosolic leucine–tRNA synthetase and catalyzes the ligation of L-leucine to tRNA (Leu). Additionally, it activates mTORC1 in a leucine-dependent manner.
40	Lrrc69	This is leucine-rich repeat-containing 69.
41	Got2	Aspartate aminotransferase is involved in amino acid metabolism and metabolite exchange between the mitochondria and cytosol. It helps the uptake of long-chain free fatty acids.
42	Rsf1	Remodeling and spacing factor 1 is a histone chaperone and, in association with several other cellular ATPases, regulates chromatin remodeling and gene expression.
43	Aco2	Aconitate hydratase is a mitochondrial aconitase/IPM isomerase family of proteins that catalyzes the biochemical reaction of the isomerization of citrate to isocitrate via cis-aconitate.
44	Nat10	RNA cytidine acetyltransferase is an RNA cytidine acetyltransferase that catalyzes the formation of N(4)-acetylcytidine in 18S rRNA.
45	Myh6	Alpha-myosin heavy chain (alpha-MHC) is primarily expressed in the cardiomyocytes. It is a major building block of sarcomeres and regulates cardiomyocyte contractility.
46	Ppif	Peptidyl-prolyl cis–trans isomerase F is a mitochondrial protein that accelerates protein folding through catalyzation of the cis–trans isomerization of proline imidic peptide bonds. Additionally, it is involved in regulating the mitochondrial permeability transition pore (mPTP).
47	Mdh2	Malate dehydrogenase is a mitochondrial protein. It plays a critical role in cellular metabolic coordination between the cytosol and mitochondria by catalyzing the reversible oxidation of malate to oxaloacetate.
48	Nnt	Nicotinamide nucleotide transhydrogenase (NNT) is an inner mitochondrial membrane protein. This enzyme couples the proton flow by hydride transfer from NAD(H) to NADP(+). Additionally, during adverse conditions, it helps maintain mitochondrial membrane potential via proton pumping.
49	Tcp1	T-complex protein 1 subunit alpha is a chaperone protein and helps in the folding of several cellular proteins such as RAP53/TCAB, actin, and tubulin.
50	Cox4i1	Cytochrome c oxidase subunit 4 isoform 1 is a mitochondrial protein that is a component of the cytochrome c oxidase and plays an important role in oxidative phosphorylation.
51	Atp5pd	ATP synthase subunit d is a mitochondrial protein in Complex V that produces ATP from ADP in the presence of a proton gradient across the membrane.
52	Myl4	Myosin light chain 4 is the regulatory light chain of myosin and an important part of sarcomeres. It helps in the cross-bridge kinetics of sarcomeres by allowing force generation.
53	Naa50	This is a highly conserved eukaryotic N-terminal acetyltransferase found to be localized in the nucleoplasm. It helps in chromosome segregation during mitosis. Additionally, it acetylates beta-tubulin.
54	Smc3	Structural maintenance of chromosome protein 3 is the central component of cohesion and plays a critical role in the cell cycle. It also takes part in DNA replication, repair, and spindle pole assembly during mitosis and in chromosome movement.
55	Pdhb	This is a subunit E1 component of the pyruvate dehydrogenase complex and localizes to mitochondria. It plays an important role in cellular metabolism by linking glycolysis and cellular ATP production via decarboxylation of pyruvate and generation of acetyl-CoA and CO_2_.
56	Slc25a4	ADP/ATP translocase 1 is involved in mitochondrial ADP/ATP transport and catalyzes the exchange of cytoplasmic ADP with mitochondrial ATP across the inner mitochondrial membrane.
57	Myl2	Myosin regulatory light chain 2 is an important component of the cardiomyocyte contractile apparatus. It plays an important role in cardiac muscle contraction through cross-bridge formation and force generation.
58	Pdhx	The pyruvate dehydrogenase complex component X is a mitochondrial noncatalytic component of PDH. It binds with subunit E3 of the PDH complex protein and regulates the conversion of pyruvate to acetyl coenzyme, linking glycolysis to the Krebs cycle.
59	Jpt1	Jupiter microtubule-associated homolog 1 negatively modulates AKT-mediated GSK3B signaling. It also regulates other cellular functions like the cell cycle and cell adhesion and inhibits AR signaling through the degradation of receptors.
60	Glud1	Glutamate dehydrogenase 1 is a mitochondrial glutamate dehydrogenase that converts L- glutamate into alpha-ketoglutarate. It plays an important role in glutamine anaplerosis by producing alpha-ketoglutarate. This is an intermediate in the tricarboxylic acid cycle.
61	Ywhaz	14-3-3 protein zeta/delta plays a critical role in cellular signaling by regulating the activity of ARHGEF7 through binding with the phosphoserine or phosphothreonine motif of the protein. It also helps in cellular maturation.
62	Myl3	Myosin light chain 3 is a regulatory light chain of myosin. It regulates the cardiac muscle contraction and muscle filament sliding. Mutation of this protein causes cardiac hypertrophy.
63	Rpl14	60S ribosomal protein L14 is a component of the large ribosomal subunit that belongs to the eukaryotic ribosomal protein eL14 family.
64	Tpm1	The tropomyosin alpha-1 chain is an actin–myosin binding protein and regulates muscle contraction through cross-bridge formation.
65	Eef1g	Elongation factor 1-gamma is an important regulatory protein of cells, which helps in the translation of protein through the delivery of aminoacyl tRNAs to the ribosome during the elongation step.
66	Vdac3	Voltage-dependent anion-selective channel protein 3 is a mitochondrial membrane protein and regulates mitochondrial diffusion of small hydrophilic molecules.
67	Cryab	Alpha-crystallin B chain is a chaperonic protein and is involved in protein folding.
68	Anxa6	Annexin A6 is a protein that may associate with CD21 and regulate the release of Ca(2^+^) from intracellular stores. This protein belongs to the annexin family.
69	Akr1b1	Aldo-keto reductase family 1 member B1 catalyzes the NADPH-dependent reduction of a wide variety of carbonyl-containing compounds to their corresponding alcohols.
70	Crat	Carnitine O-acetyltransferase acts as a catalyst for the reversible transfer of acyl groups from carnitine to coenzyme A and regulates the ratio of acyl-CoA/CoA. It also plays a crucial role in the transport of fatty acids for beta-oxidation and it may be specific for short-chain fatty acids.
71	Mrpl47	Mitochondrial ribosomal protein L47 plays an important role in mitochondrial translational and metabolism of proteins.
72	Hspe1	This is a 10 kDa heat shock protein that is in the mitochondria. It is a co-chaperonin implicated in mitochondrial protein import and macromolecular assembly. It coordinates with Hsp60 and facilitates the correct folding of imported proteins. Additionally, during cellular stress, this protein binds with the unfolded protein and helps in proper folding.
73	Ak3	GTP:AMP phosphotransferase AK3 is a mitochondrial protein that catalyzes the interconversion of nucleoside phosphates and is involved in maintaining the homeostasis of cellular nucleotides. It performs GTP to AMP phosphotransferase and ITP to AMP phosphotransferase activities.
74	Rbbp4	RB-binding protein 4 is a chromatin-remodeling factor. It promotes the repression of gene expression through binding with several chromatin regulatory factors including histone deacetylases.
75	H2az1	Histone H2A.Z is a histone protein that regulates DNA folding and gene transcription.
76	Lgals1	Galectin-1 is a lectin that binds beta-galactosidase and a different class of carbohydrates. It plays an important role in the different cellular functions including apoptosis, cell proliferation, and cell differentiation. This protein helps to maintain the phosphorylation of Lyn kinase by inhibiting the phosphatase activity of CD45.
77	Rrbp1	Ribosome-binding protein 1 is an endoplasmic reticulum protein and helps in ER proliferation, secretion, and cell differentiation.
78	Dld	Dihydrolipoyl dehydrogenase is a mitochondrial protein and part of three enzymes: (i) branched-chain alpha-ketoacid dehydrogenase complexes (BCKDH), (ii) alpha-ketoacid dehydrogenase complexes (αKGDH), and (iii) pyruvate dehydrogenase (PDH) complex. It mainly regulates cellular energy metabolism as well as lysine succinylation of histones in the nucleus.
79	Myh9	Myosin-9 plays an important role in cytoskeleton reorganization and regulates cell division, shape, and secretion.
80	Shmt2	Serine hydroxymethyltransferase regulates the interconversion of serine and glycine and belongs to the SHMT family.
81	Hadha	Trifunctional enzyme subunit alpha is a mitochondrial enzyme that catalyzes the last three reactions in the mitochondrial beta-oxidation pathway. It helps in the energy production of the tissue through mitochondria by helping in the four consecutive breaking down reactions of fatty acids into acetyl-CoA.
82	Echs1	Enoyl-CoA hydratase is a mitochondrial enzyme. Straight-chain enoyl-CoA thioesters from C4 to at least C16 are processed by decreasing the catalytic rate. This enzyme acts on substrates like crotonyl-CoA, acryloyl-CoA, 3-methylcrotonyl-CoA, and methacrylyl-CoA.
83	Sh2d3c	This protein contains a guanine nucleotide exchange factor-like domain, which binds with the Ras family of GTPases and acts as an adaptor protein. It is involved in cell migration.
84	Ywhaq	14-3-3 protein theta is an adapter protein that regulates the kinase activity of PDPK1.
85	Uqcrc2	Cytochrome b-c1 complex subunit 2 is a mitochondrial protein and regulates mitochondrial electron transport chain reaction through the formation of the ubiquinol–cytochrome c reductase complex (complex III).
86	Rabepk	This is a Rab9 effector protein having kelch motifs. It is involved in receptor-mediated endocytosis and vesicle docking, which are involved in exocytosis.
87	Sox7	SRY-box containing gene 7 acts as a transcription factor. It forms complex with other cellular proteins and regulates embryonic development and cellular fate.
88	Cep295	The centrosomal protein of 295 kDa is a centriole-enriched microtubule-binding protein. This protein is localized in the cytosol microtubule and plasma membrane and positively regulates protein acetylation.
89	Myl6	Myosin light polypeptide 6 is a regulatory light chain of myosin. This protein plays a key role in cellular movement by helping in force generation. Additionally, it is involved in muscle contraction, platelet activation, cell viability, vesicle-mediated cargo transport, endocytosis, and cancer cell progression.
90	Lrif1	This protein belongs to the LRIF1 family. It localizes to the centriolar satellite, nucleoplasm, chromosome, telomeric region, and nuclear lumen. In females, it acts as a repressor of chromosome X. Additionally, it represses the function of retinoic acid alpha through direct recruitment of histone deacetylase.
91	Il16	Pro-interleukin-16 stimulates a migratory response in CD4+ lymphocytes, monocytes, and eosinophils. It also primes CD4+ T-cells for IL-2 and IL-15 responsiveness and induces T-lymphocyte expression of interleukin 2 receptor. It is a ligand for CD4.
92	Gtf3c1	General transcription factor 3C polypeptide 1 is an important component of RNA polymerase and regulates transcription.
93	Rps9	40S ribosomal protein S9 belongs to the universal ribosomal protein uS4 family. This protein plays an important role in ribosome biogenesis, translation, cell growth and proliferation, apoptosis, DNA repair, and developmental regulations.
94	Eci2	Enoyl-CoA delta isomerase 2 is a mitochondrial enzyme that is able to isomerize both 3-cis and 3-trans double bonds into the 2-trans form in a range of enoyl-CoA species. Additionally, it is involved in lipid metabolism and promotes cancer cell survival.
95	Arhgap20	Rho GTPase-activating protein 20 is a GTPase activator for the Rho-type GTPases by converting them to an inactive GDP-bound state.
96	Tmem176b	Transmembrane protein 176B is required for the development of cerebellar granule cells and it may regulate the maturation of dendritic cells.
97	Tspy1	Testis-specific Y-encoded protein is involved in cellular metabolism such as spermatogenesis, cell proliferation, and androgen signaling.
98	Cinp	This is potentially similar to the cyclin-dependent kinase 2-interacting protein, isoform CRA_a. This protein regulates ATR-dependent signaling, resistance to stress, and G2 checkpoint integrity.
99	Spag17	Sperm-associated antigen 17 is associated with several cellular functions such as germ cell differentiation, bone development, structure and motility of cilia, and nuclear translocation of protamines.
100	Angptl3	Angiopoietin-like 3 isoform CRA_b plays an important role in lipoprotein metabolism.
101	Mrpl3	39S ribosomal protein L3 localizes to mitochondria and belongs to the universal ribosomal protein uL3 family. It is involved in the biosynthesis of mitochondrial protein and the structure and biogenesis of ribosomes.

## Data Availability

The material and data associated with the work will be available upon request from the corresponding author.

## Supplementary Materials

The following supporting information can be downloaded at: https://www.mdpi.com/article/10.3390/metabo14120701/s1, Figure S1: Experimental Workflows, and Figure S2: The pie chart shows the relative distribution of the acetylated proteins identified by mass spectroscopy in percentage.
