# Supplementary material for: Acetylation-Mediated Post-Translational Modification of Pyruvate Dehydrogenase Plays a Critical Role in the Regulation of the Cellular Acetylome During Metabolic Stress
Source: Metabolites. 2024 Dec 12;14(12):701. doi: 10.3390/metabo14120701 (PMC11679536; doi:10.3390/metabo14120701)
Supplement: Supplementary file 1 [file metabolites-14-00701-s001.zip › metabolites-3313028-supplementary.pdf]

Supplementary Figure 1:

Experimental Workflows:

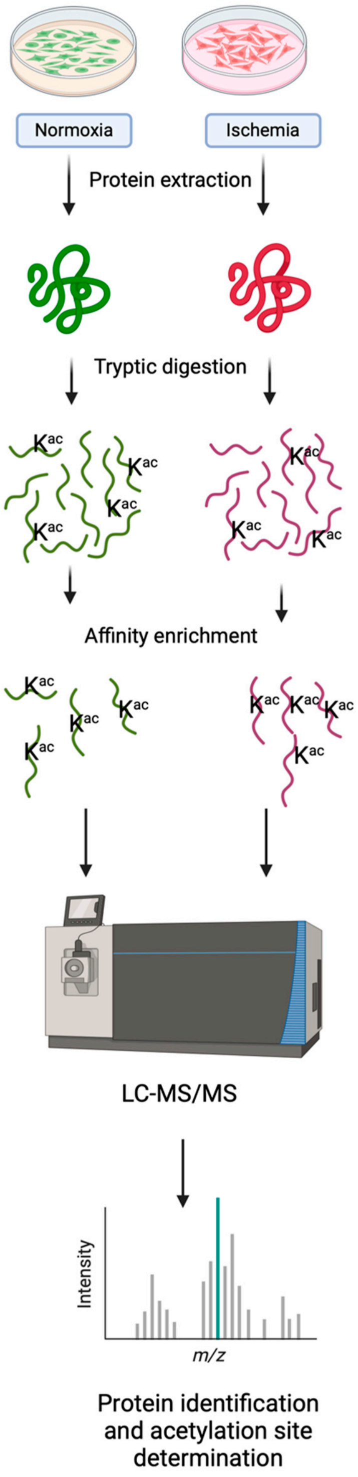

Supplementary Figure 2:

Protein localization:

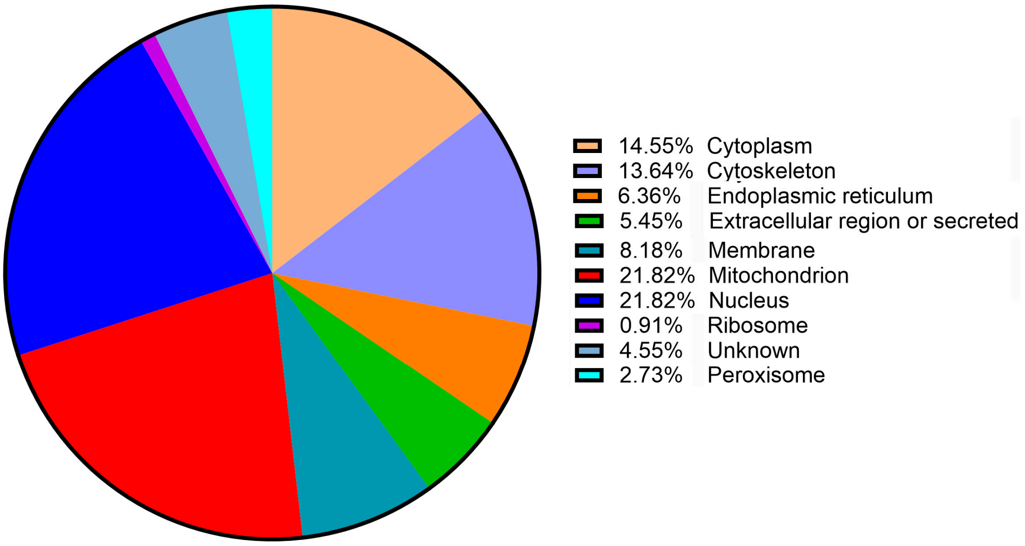

Supplementary Figure 2: The pie chart shows the relative distribution of the acetylated proteins identified by mass spectroscopy in percentage.
